# Efficacy and Tolerability of Metyrapone in Mild Autonomous Cortisol Secretion: Real‐World Findings From Clinical Practice

**DOI:** 10.1111/cen.70056

**Published:** 2025-11-06

**Authors:** Simon Berry, Ahmed Iqbal, John Newell‐Price, Miguel Debono

**Affiliations:** ^1^ Division of Clinical Medicine University of Sheffield Sheffield UK; ^2^ Department of Diabetes and Endocrinology Sheffield Teaching Hospitals NHS Foundation Trust Sheffield UK

**Keywords:** adrenal incidentaloma, MACE, MACS, metyrapone

## Abstract

**Objective:**

Mild autonomous cortisol secretion (MACS) is associated with increased cardiometabolic risk factors including hypertension, type 2 diabetes and dyslipidaemia. By using evening doses of metyrapone, a short‐acting 11‐β hydroxylase inhibitor, it has been shown that it is possible to reset the abnormal circadian cortisol rhythm in MACS. This study aimed to evaluate the tolerability and impact of this approach on cardiometabolic outcomes in patients with MACS.

**Design:**

We conducted a single‐centre retrospective, longitudinal review of patients with MACS who received evening metyrapone (250–500 mg at 6 PM and 250 mg at 10 PM) to evaluate adverse events, tolerability, and cardiometabolic outcomes (systolic and diastolic blood pressure, HbA1c, weight and non‐HDL cholesterol) at 6 months, compared to controls. Age and sex‐matched controls were identified from patients with adrenal incidentalomas and non‐suppressed serum cortisol following 1 mg overnight dexamethasone suppression testing.

**Results:**

Fifteen patients and 15 matched controls were identified. Over 6 months there were no adrenal crises. Metyrapone was stopped in 2/15 patients in view of side effects. In the metyrapone group compared to controls, there were significant decreases in systolic blood pressure (−17.7 (SE 5.8) vs. +8.7 (5.7)mmHg, *p* = 0.008, *n* = 9) and diastolic blood pressure (−9.9 (4.2) vs. +3.0 (3.6)mmHg, *p* = 0.024). The differences between groups for HbA1c, weight and non‐HDL cholesterol were not statistically significant.

**Conclusion:**

Evening metyrapone was associated with significant reductions in systolic and diastolic blood pressure in patients with MACS, without causing adrenal insufficiency, indicating its potential safe clinical utility. A well‐powered, controlled, prospective study is needed to validate these findings and comprehensively investigate the broader metabolic outcomes.

**Abbreviations**
ACTH: adrenocorticotropic hormone
DBP: diastolic blood pressure
HbA1c: haemoglobin A1c
HR: hazard ratio
IQR: interquartile range
MACS: mild autonomous cortisol secretion
ONDST: overnight dexamethasone suppression test
RR: relative risk
SBP: systolic blood pressure
SD: standard deviation
SE: standard error

## 1 Introduction

Adrenal incidentalomas are commonly found on axial imaging [1]. The prevalence of adrenal incidentalomas is less than 1% of those aged under 20 but increases to around 10% of those aged 70 years. Around 30%–50% of adrenal incidentalomas exhibit autonomous cortisol secretion [2]. Mild autonomous cortisol secretion (MACS) is defined as a post dexamethasone serum cortisol above 50 nmol/L (1.8 µg/dL) [1]. It is characterised by cortisol excess, particularly in the nocturnal period [3, 4, 5]. MACS is associated with a higher prevalence of type 2 diabetes, hypertension, dyslipidaemia and frailty [6]. Furthermore, MACS confers a two‐fold higher risk of all‐cause mortality compared to matched controls [7]. The risk is increased in those aged under 65, with further increases in risk directly correlating with higher post dexamethasone serum cortisol levels [7, 8]. More recent data suggest that MACS may also lead to osteoporosis and increased fracture risk [9, 10]. The European Society of Endocrinology and ENSAT Guidelines from 2023 recommend discussion of the option of adrenalectomy for patients with MACS where there is a unilateral adrenal adenoma in addition to relevant comorbidities, taking account of individual factors and patient preference [1]. Adrenalectomy for MACS has been demonstrated to increase the chances of improvement in blood pressure, glucometabolic control and dyslipidaemia; reduce vertebral fractures, and improve quality of life when compared to conservative management [6, 11, 12]. With the increasing incidence of adrenal incidentalomas with MACS, it will not be feasible to offer an adrenalectomy to every patient, and it is not always a suitable treatment when there are bilateral adenomas, or where surgery is risky or not acceptable to the patient. Alternatively, MACS can be managed conservatively with management of cardiometabolic risk factors such as hypertension, dyslipidaemia and type 2 diabetes [13]. However, this treatment does not treat the root‐cause of these issues, the cortisol excess, and has been shown to be inferior compared to adrenalectomy in controlling cardiovascular markers [11, 12]. There remains an unmet need to develop a medical treatment for MACS. Metyrapone is an 11‐β hydroxylase inhibitor with a short duration of action [14]. In a previous study, we demonstrated that the use of timed evening doses of metyrapone (500 mg at 6 PM and 250 mg at 10 PM) resets the abnormal nocturnal cortisol rhythm without affecting morning and daytime cortisol levels, leading to a reduction in the cardiovascular risk marker, IL‐6 [3]. Here, we hypothesised that administration of long‐term evening metyrapone would reduce cardiometabolic dysfunction in patients with MACS. In this real‐world retrospective study, we therefore investigated the tolerability of evening metyrapone and its effects on cardiometabolic risk factors in patients with MACS treated at our university hospital.

## 2 Materials and Methods

A retrospective, longitudinal study was carried out to assess the tolerability and clinical outcomes of all patients who received metyrapone treatment as part of routine clinical care, 250–500 mg at 6 PM and 250 mg at 10 PM, for MACS at Sheffield Teaching Hospitals NHS Foundation Trust (Sheffield, United Kingdom) between January 2016 and January 2023. The usual starting dose was 500 mg at 6 PM and 250 mg at 10 PM but a lower dose of 250 mg at 6 PM and 10 PM was used if there was increased frailty. If intolerable side effects were experienced on the higher dose, morning serum cortisol levels were checked, metyrapone was suspended for 2 weeks and then re‐started at 250 mg at 6 PM and 10 PM. If side effects were persistent, metyrapone was re‐started at 250 mg at 6 PM only. Cases were identified and patient data extracted from the electronic medical records system. All patients referred to endocrinology via the adrenal incidentaloma pathway underwent an overnight dexamethasone suppression test (ONDST). The ONDST protocol involved self‐administration of 1 mg oral dexamethasone at 23:00, followed by blood sampling for cortisol and adrenocorticotropic hormone (ACTH) at 0800–0900 h the next morning. Those with a non‐suppressed serum cortisol ( > 50 nmol/L, > 1.8 ug/dL) were identified as having MACS. Metyrapone was prescribed off‐license for a subset of these patients based on the following criteria: a diagnosis of MACS, suppressed ACTH < 10 ng/L, and hypertension or diabetes mellitus requiring escalation of treatment. Those started on metyrapone either had bilateral adenomas or were patients with unilateral adenomas who declined or were not fit for surgery. Patients were included by consecutive sampling. Age and sex‐matched controls were identified from adrenal incidentaloma referrals, during the same time period, who did not meet the criteria for metyrapone or adrenalectomy. The study was approved as an Institutional Case Notes review by Sheffield Teaching Hospitals NHS Foundation Trust (Project Reference Number 11604).

### 2.1 Patient Instructions

Patients initiating metyrapone therapy were instructed to administer the medication orally with a glass of milk or a small snack to minimise gastrointestinal upset, and were informed about potential adverse effects, such as dizziness. They were advised to administer 10 mg oral hydrocortisone every 6 h in case of acute illness and recommended extra steroid cover for any surgery. Steroid cards and emergency hydrocortisone packs were provided. Contact information for an endocrine specialist nurse was provided in case of need for further guidance.

### 2.2 Post‐Initiation Monitoring

Two weeks after initiation of evening metyrapone, a morning serum cortisol was performed to monitor for adrenal over‐suppression, and analysed by the Elecsys Cortisol II assay (*Roche* Diagnostics GmbH, Mannheim, Germany) (Cobas interassay precision coefficient of variation (CV) 1.1%–5.5% at serum cortisol 3.62–1660 nmol/L). In female patients, DHEAS and testosterone levels were monitored on an ad‐hoc basis.

### 2.3 Outcome Measures

The outcomes identified before the review was conducted were tolerability at 6 months (defined as continuing to take metyrapone at 6 months) and change in systolic (SBP) and diastolic blood pressure (DBP), weight, HbA1c and non‐HDL cholesterol between baseline and 6 months. Non‐HDL cholesterol was chosen as the pre‐specified marker of dyslipidaemia as it is recommended by the National Institute for Health and Care Excellence (UK) for monitoring treatment response in primary prevention of cardiovascular disease and is consistently available on all locally analysed lipid profiles (both fasting and random) [15]. Electronic notes were reviewed for any side effects reported by patients at clinic, for hospitalisations and emergency department attendances, for ongoing metyrapone prescription and for measurements of weight. The electronic laboratory results systems were screened for HbA1c, lipid profiles, ACTH and morning serum cortisol results. Blood pressure was checked in clinic in the sitting position after at least 5 min resting at baseline and at a routine follow‐up clinic visit. In all patients where MACS was found, an advice letter was sent to the primary care provider advising monitoring and management of hypertension, diabetes, and dyslipidaemia to reduce long term cardiovascular risk.

### 2.4 Statistical Methods

Statistical analysis was performed with IBM SPSS Statistics Version 29 (Chicago, USA). Baseline characteristics are summarised per group by descriptive statistics using number of patients (n) for categorical variables, and median and interquartile range (IQR) for continuous variables. Morning serum cortisol levels post‐metyrapone initiation are summarised as median and IQR. Wilcoxon signed rank test was used for paired analysis of metabolic outcomes pre‐ and post‐treatment. A Mann–Whitney *U* test was used to compare differences in baseline characteristics between the metyrapone group and control group. Additionally, a Mann–Whitney *U* test was applied to compare the 6‐month change from baseline in the metyrapone group against that of the 6‐month change from baseline in the control group. In parameters in which follow‐up data was missing, paired baseline data were also omitted from analysis.

## 3 Results

### 3.1 Baseline Characteristics

Fifteen patients who had been initiated on metyrapone for MACS during the study period were identified, along with 15 age‐ and sex‐matched controls. Baseline characteristics are displayed in Table 1, with further detail of individual characteristics in Supporting Information S1: Table 1. Baseline cortisol post‐ONDST (*p* = 0.001) and non‐HDL cholesterol (*p* = 0.005) were significantly higher in the metyrapone group compared to the control group. HbA1c, SBP, DBP and weight were also higher in the metyrapone group but the differences did not reach statistical significance.

**Table 1:** Baseline characteristics of control and metyrapone groups, presented as median (IQR). Statistical comparison between groups by Mann–Whitney *U* test.

Characteristic	Control	Metyrapone	*p* value
Age	66 (61.5–77)	67 (60.5–76)	0.967
Gender			
Male	4	4	
Female	11	11	
Location			
Bilateral	5	9	
Unilateral	10	6	
Cortisol post ONDST (nmol/L)	74.0 (57.5–88.0)	125.0 (99.0–142.5)	0.001**
ACTH (ng/L)	7.5 (6–9)	4.5 (3–7.3)	0.410
Weight (kg)	84.0 (68.1–97.8)	95.0 (79.5–114.8)	0.165
HbA1c (mmol/mol)	42.0 (38.5–62.0)	45.5 (41.5–57.0)	0.684
Non‐HDL cholesterol (mmol/L)	2.2 (1.7–2.9)	3.5 (2.9–4.1)	0.005**
Systolic blood pressure (mmHg)	131 (118–147)	140 (139–153)	0.190
Diastolic blood pressure (mmHg)	70 (69–81)	84 (78–87)	0.136

Abbreviations: HbA1c, haemoglobin A1c; ONDST, overnight dexamethasone suppression test.

** *p* < 0.01.

### 3.2 Safety Profile

Morning serum cortisol levels were measured following evening metyrapone. The median morning serum cortisol level was 365 nmol/L (13.2 µg/dL) (IQR 254–431 nmol/L (9.2–15.6 µg/dL)). Median ACTH increased from 4.5 to 7.5 ng/L (*n* = 12, *p* = 0.032) after treatment for 6 months.

Metyrapone was stopped in 2/15 patients in view of side effects—one stopped due to diarrhoea after 1 week, and one due to asymptomatic persistently raised serum testosterone and DHEAS levels in a woman, first noted within 1 month of commencing treatment (Table 2). Further details of side effects and symptom onset are provided in Supporting Information S1: Table 1. There were no adrenal crises in the 6‐month follow‐up period. There was one hospitalisation, unrelated to metyrapone, due to acute‐on‐chronic hyponatraemia secondary to psychogenic polydipsia. All other side effects were transient and tolerated. There were two other ambulatory care attendances, unrelated to metyrapone: an ankle injury and a natal cleft infection.

**Table 2:** A list of all side effects reported after starting metyrapone (*n* = 15).

Side effects reported	No. of patients
Adrenal crisis	0
Diarrhoea	1
Dizziness	3
Headaches	1
Hyperandrogenism in a woman	1
Nausea	2

### 3.3 Cardiometabolic Outcomes

**Figure 1:**
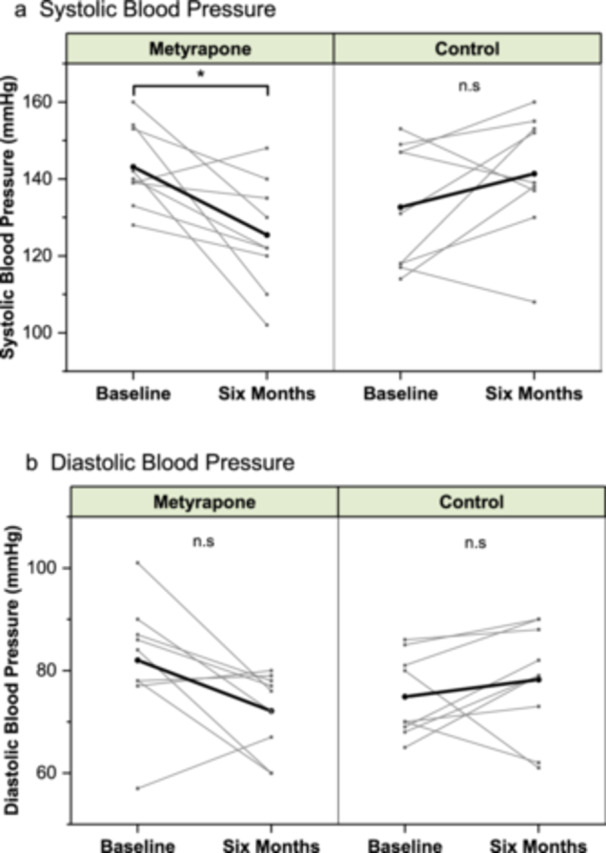
(a) Systolic blood pressure (SBP) and (b) diastolic blood pressure (DBP) at baseline and at 6 months in metyrapone and control groups (*n* = 9). Each grey line represents an individual participant and the black lines represent the mean. *p < 0.05, n.s., nonsignificant.

**Figure 2:**
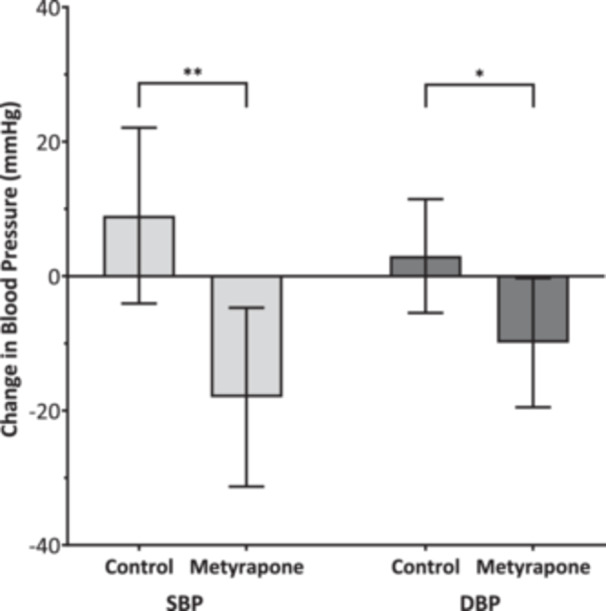
Bar chart of change in mean systolic blood pressure (SBP) and diastolic blood pressure (DBP) following treatment (*n* = 9 in each arm) (95% CI error bars). Significant decrease in SBP (*p* = 0.008) and DBP (*p* = 0.024) with metyrapone compared to controls. *p < 0.05, **p < 0.01.

In the metyrapone group at 6 months, there was a significant absolute decrease in SBP (17.7 mmHg, SE ± 5.8; *p* = 0.021, *n* = 9) compared to baseline (Figure 1). At baseline, three of nine patients had a SBP ≤ 140 mmHg and four of nine had a DBP ≤ 80 mmHg. By follow‐up, the proportion reaching the target had increased, with seven of nine patients achieving SBP control and all nine achieving DBP control. When comparing the changes in blood pressure from baseline to 6 months to controls, the metyrapone group showed significant decreases in SBP (−17.7 (SE 5.8) vs. +8.7 (5.7) mmHg; *p* = 0.008), and DBP (−9.9 (4.2) vs. +3.0 (3.6) mmHg; *p* = 0.024) (Figure 2). Between measurements, four patients in the control group received additional new antihypertensive medication, compared to only one in the metyrapone group (Supporting Information S2: Table 2). There was no significant difference in the change from baseline to 6 months for HbA1c, weight, or non‐HDL cholesterol between the metyrapone and control groups (Table 3). While the mean increase in HbA1c over this period was numerically lower in the metyrapone group (+0.2 mmol/mol) compared to controls (+3.3 mmol/mol), resulting in an absolute difference of 3.1 mmol/mol (SE ± 4.5, *n* = 10), this finding did not reach statistical significance. Similarly, non‐HDL cholesterol increased by 0.4 mmol/L in the control group after 6 months but there was no change in the metyrapone group (SE ± 0.33, *n* = 10). In contrast, the weight increased by 0.2 kg in the metyrapone group after 6 months, compared to a 0.7 kg weight loss in the control group (SE ± 3.9, *n* = 10).

**Table 3:** Table showing cardiometabolic markers (mean) at baseline, at 6 months, and the absolute difference at 6 months compared to baseline, for control and metyrapone groups (*n* = 10). Before and after, and between group comparisons did not demonstrate statistical significance. Reference ranges: HbA1c 19‐47 mmol/mol; non‐HDL cholesterol N/A.

	Control			Metyrapone		
	Baseline	Six months	Difference	Baseline	Six months	Difference
Weight (kg)	83.9	83.2	−0.7	98.2	98.4	+0.2
HbA1c (mmol/mol)	51.4	54.7	+3.3	52.1	52.3	+0.2
Non‐HDL Cholesterol (mmol/L)	2.3	2.7	+0.4	3.7	3.7	0.0

Abbreviation: HbA1c, haemoglobin A1c.

## 4 Discussion

We present a real‐world retrospective study that has demonstrated that timed evening metyrapone administration in MACS led to a clinically and statistically significant reduction in blood pressure. Importantly, morning serum cortisol was not low on treatment and there were no adrenal crises. This study shows that metyrapone can address at least one of the cardiometabolic risk factors associated with MACS, supporting its potential clinical utility. In contrast, within the confines of a study with limited numbers, there were no significant differences in weight, non‐HDL cholesterol or HbA1c in those treated with metyrapone. Rigorous up‐titration of antihypertensives would be an alternative to treatment with metyrapone but it is notable that the MACS group on metyrapone had a significantly greater reduction in systolic and diastolic blood pressure despite more patients in the control group receiving additional new antihypertensive medication within the study period, and those on metyrapone having a higher degree of hypercortisolaemia as reflected by higher post‐dexamethasone serum cortisol. Among studies assessing adrenalectomy for MACS, the CHIRACIC trial similarly showed a benefit limited to blood pressure, whereas the COAR study observed broader improvements in weight, glycaemia, and blood pressure [11, 12].

The goal of treatment with metyrapone is to reset the nocturnal cortisol rhythm without causing adrenal insufficiency in the daytime. One advantage of metyrapone is the short 4‐h half life of the active metabolite. Previously we showed that metyrapone can acutely reset the nocturnal cortisol rhythm without affecting morning cortisol levels [3]. Here, we now demonstrate that such an approach is feasible over the long term, is safe and appears to be effective. The side effects experienced by patients taking metyrapone in this study were generally well tolerated and transient, except for one patient who stopped metyrapone due to diarrhoea and one due to asymptomatic hyperandrogenism in a woman. Gastrointestinal upset is a known side effect of metyrapone and in a multi‐centre retrospective study of metyrapone in Cushing's disease, was observed in 23% of participants [16]. Due to its mechanism of action as an 11β hydroxylase inhibitor, androgenic steroid intermediates may accumulate, presenting with acne or hirsutism in women [17]. The required doses to manage hypercortisolism in MACS are much lower compared to Cushing's syndrome. Although accumulation is less probable when cortisol secretion is autonomous, extra caution would be advisable with chronic metyrapone use in women [18].

Current clinical practice is to consider adrenalectomy for people with unilateral adrenal adenomas with MACS and resultant comorbidites [1] but adrenal incidentalomas with MACS are common [19] and it is not feasible to offer an adrenalectomy to every patient. It might not be a suitable treatment when there are bilateral adenomas, or where surgery is not feasible or acceptable to the patient. Conservative management appears to be inferior and does not address bone health or any other metabolic effects the cortisol excess may cause [6, 11, 12]. The results of our study suggest that metyrapone may fill this gap, by directly tackling the cortisol excess, and thus addressing its consequences. Theoretically, medical management might also be useful as a bridge to surgery or even to stratify patients who will gain most benefit from surgery. Surgical data show that there are people with MACS whose cardiometabolic outcomes respond to surgery and a significant proportion in which they do not [6]. If cardiometabolic improvements were demonstrated after starting medical treatment, one could hypothesise that they would have an increased chance of responding to surgery. There are other medications already in clinical use to treat cortisol excess caused by overt Cushing's syndrome, including mitotane, mifepristone, osilodrostat and ketoconazole [18]. Mifepristone, an oral non‐selective glucocorticoid receptor antagonist, has been shown to reduce insulin resistance in some individuals with MACS [20, 21], and improve glycaemia in people with poorly controlled type 2 diabetes and hypercortisolism (with or without an adrenal adenoma) [22]. There has also been a case series in which mifepristone and ketoconazole have been used to treat bilateral macronodular adrenal hyperplasia [23, 24]. Yet, to our knowledge, there have been no formal published randomised controlled clinical trials of any of these agents for MACS, and so there are no data for comparison.

Our study is limited by low patient numbers. Further prospective, adequately‐powered studies with a larger population would be required to investigate for effects on the other cardiometabolic risk factors. Although the control group was selected from the same population group, the patients within the metyrapone group had been treated medically for MACS based on greater clinical need and therefore, the baseline characteristics are different, particularly the higher cortisol levels post‐ONDST. Therefore, it is likely that the metyrapone‐treated group had co‐morbidities more likely to be driven by MACS rather than a bystander to MACS. Larger studies are needed to assess the side effect profile of the lower doses of metyrapone used here, particularly in females for whom hyperandrogenism is a potential concern. Ideally, a study using cardiovascular outcomes, rather than surrogate markers, would be performed but realistically, the required follow‐up period to determine a meaningful difference would be too long to be feasible at this stage. This study was retrospective and conducted in the real‐world without a standardised titration schedule for antihypertensive medications. Specifically, blood pressure readings were conducted in clinic and titration of antihypertensives was delegated to the primary care provider, who are advised by the local integrated care board to use the NICE NG136 Guideline but prescription changes were as per their own clinical discretion [25]. While this potentially introduces bias, it also means that findings reflect effectiveness and generalisability in routine clinical practice. Similarly, clinic blood pressure readings do not always reflect long term blood pressure control, and use of home blood pressure readings would strengthen future studies.

In conclusion, evening metyrapone is associated with significant reductions in systolic and diastolic blood pressure in patients with MACS, supporting its potential clinical utility to reduce cardiometabolic dysfunction. It did not cause any adrenal crises but monitoring early morning cortisol is essential to avoid over‐treatment. This small retrospective study, albeit limited, provides data to inform a larger, controlled, prospective clinical trial.

## Conflicts of Interest

J.N.P has received research income and consultancy from Crinetics and Sparrow, which was paid to the University of Sheffield. The University of Sheffield holds IP in relation to the use of medical treatment for MACS. M.D. has received honorarium from Esteve. The other authors declare no relevant conflicts of interest.

## Supporting information

Supplementary Table 1 V1.

Supplementary Table 2.
