# Supplementary material for: Efficacy and Tolerability of Metyrapone in Mild Autonomous Cortisol Secretion: Real‐World Findings From Clinical Practice
Source: Clin Endocrinol (Oxf). 2025 Nov 6;104(3):215–21. doi: 10.1111/cen.70056 (PMC12865743; doi:10.1111/cen.70056)
Supplement: Supplementary file 1 — Supplementary Table 1 V1. [file CEN-104-215-s002.pdf]

Patient	Age	Sex	Bilateral or Unilateral adenoma	Declined or not fit for surgery	Metyrapone Regimen	Ongoing prescription at 6 months	Side effects	Side effect onset (days post start)	Baseline Blood Pressure	6-month Blood Pressure
1	52	Male	Bilateral	N/A	500mg at 1800 + 250mg at 2200	No	None (stopped due to psychogenic polydipsia)	-	N/A	N/A
2	83	Female	Bilateral	N/A	250mg at 1800 + 250mg at 2200[a]	Yes	None	-	153/90	140/72
3	67	Male	Bilateral	N/A	250mg at 1800 + 250mg at 2200	No	Diarrhoea	7	N/A	N/A
4	30	Female	Unilateral	Opted for surgery at later date	250mg at 1800 (reduced due to s/e)	Yes	Dizziness, nausea (improved after stopping metyrapone, did not recur on reduced dose)	3	133/86	122/77
5	64	Female	Bilateral	N/A	250mg at 1800 + 250mg at 2200	Yes	None	-	142/87	102/78
6	66	Female	Unilateral	Opted for surgery at later date	500mg at 1800 + 250mg at 2200	Yes	None	-	139/78	148/79
7	85	Female	Bilateral	N/A	250mg at 1800 + 250mg at 2200	Yes	Dizziness (exacerbation of pre-existing symptom)	28	125/60	Missing data
8	77	Female	Bilateral	N/A	250mg at 1800 (reduced due to s/e)	No	Asymptomatic high testosterone and DHEAS (persisted after stopping metyrapone, remained asymptomatic)	28	N/A	N/A
9	75	Male	Bilateral	N/A	500mg at 1800 + 250mg at 2200	Yes	None	-	130/72	Missing data
10	68	Female	Bilateral	N/A	250mg at 1800 + 250mg at 2200	Yes	Dizziness, headaches (resolved after reducing dose)	3	140/78	122/60

11	54	Female	Unilateral	Yes	500mg at 1800 + 500mg at 2200	Yes	None	-	128/57	120/67
12	77	Female	Unilateral	Yes	250mg at 1800 + 250mg at 2200[a]	Yes	None	-	154/84	110/60
13	57	Female	Unilateral	Opted for surgery at later date	500mg at 1800 + 250mg at 2200	Yes	None		Missing data	146/76
14	75	Male	Unilateral	Yes	500mg at 1800 + 250mg at 2200	Yes	None		160/101	130/76
15	65	Female	Bilateral	N/A	500mg at 1800 + 250mg at 2200	Yes	None		139/77	135/80

Supplementary Table 1: Individual data on patients receiving metyrapone. S/e: side effect.

[a]Started directly on 250mg at 1800 and 250mg at 2200 daily due to frailty.
