# Supplementary material for: Efficacy and Tolerability of Metyrapone in Mild Autonomous Cortisol Secretion: Real‐World Findings From Clinical Practice
Source: Clin Endocrinol (Oxf). 2025 Nov 6;104(3):215–21. doi: 10.1111/cen.70056 (PMC12865743; doi:10.1111/cen.70056)
Supplement: Supplementary file 2 — Supplementary Table 2. [file CEN-104-215-s001.pdf]

| Patient number | Metyrapone Group | Control Group |
|---|---|---|
| 2 | Nil changes | Furosemide started<br>Bisoprolol started<br>Bendroflumethiazide stopped |
| 4 | Nil changes | Ramipril started |
| 5 | Nil changes | Amlodipine started |
| 6 | Indapamide stopped<br>Doxazosin stopped<br>Ramipril started | Nil changes |
| 10 | Nil changes | Nil changes |
| 11 | Nil changes | Nil changes |
| 12 | Nil changes | Bisoprolol started<br>Furosemide started<br>Candesartan reduced |
| 14 | Nil changes | Nil changes |
| 15 | Nil changes | Nil changes |

**Supplementary Table 2:** Changes in antihypertensive medications on patients receiving metyrapone and their controls during the six-month study period. All changes were made by primary care providers and hospital providers from other specialties.
